# Metal-polyphenol nanomedicines for malignant tumor therapy

**DOI:** 10.3389/fchem.2026.1834451

**Published:** 2026-05-28

**Authors:** Zhiqi Li, Hua Xin, Jincan Chen, Jiayu Dai, Hongcheng Zheng, Shuyuan Shi, Hongbin Sun, Wanpeng Lu, Gengxue Wang, Zhengjia Liu, Nan Zhao

**Affiliations:** 1 Department of Thoracic Surgery, China-Japan Union Hospital of Jilin University, Changchun, China; 2 Department of Thoracic Surgery, Gongzhuling Central Hospital, Gongzhuling, China

**Keywords:** metal-polyphenol, nanomedicines, polyphenol, synergistic therapeutic nanoplatform, tumor therapy

## Abstract

Malignant tumor is still one of the most critical diseases in clinic. Current therapeutic strategies include surgery, chemoradiotherapy, targeted therapy, and immunotherapy. Nevertheless, the development of novel drugs with superior efficacy and reduced drug toxicity remains a goal for researchers. The rise of nanomedicine has injected new momentum into oncology treatment. Among nano-platforms, metal-polyphenol materials can be engineered into nanodots, nanospheres or network structures, which can encapsulate or load metabolic enzyme inhibitors and chemotherapeutics, enabling precision therapy through both passive and active targeting. Furthermore, these metal-polyphenol nanocomposites frequently integrate the functionalities of chemodynamic therapy, photothermal therapy and photodynamic therapy, synergistically amplifying antitumor effects. To date, however, curcumin, tannic acid, and epigallocatechin gallate have dominated the metal-polyphenol nanomaterials, whereas complex metal-polyphenol nanomaterials remain scarce. Accordingly, this review systematically summarizes the advantages and mechanisms of metal-polyphenol systems categorized by metal species, dissects the antitumor mechanisms of polyphenols, and outlines the substantial potential of metal-polyphenol nanomaterials for treating malignancies.

## 1 Introduction

Based on GLOBOCAN 2020 data, this article outlines the global cancer burden. In 2020, there were approximately 19.3 million new cancer cases and nearly 10 million deaths worldwide. Breast cancer surpassed lung cancer as the most common malignancy for the first time. Lung cancer remained the leading cause of cancer death. The global cancer burden is projected to increase by 47%. By 2040, new cases will reach 28.4 million (Sung et al., 2021). The current situation indicates that the treatment of malignant tumors has become extremely urgent. More therapeutic approaches are needed to combat cancer collaboratively. Nanomaterials represent a crucial component in this effort. Metal-polyphenol nanomedicines belong to the family of nanomaterials. As natural compounds, polyphenols have attracted significant research attention and hold great promise for cancer treatment.

Metal ions are indispensable for normal cellular metabolism and signaling function, including membrane excitability, signal transduction, metalloprotein catalysis and cell death. Especially, they also play pivotal roles in tumorigenesis, tumor progression, and immune responses (Wang et al., 2020). Even the subtle shift in their intracellular concentrations can impair metabolic function and disrupt metal homeostasis (Bird, 2015; Leslie et al., 2019). Since the late 1980s, the metals have been recognized as key players in cancer processes, such as uncontrolled proliferation, evasion of apoptosis, tissue invasion and metastasis, so the metals spur intense interest in oncotherapy (Boonstra et al., 1981; Martial, 2016; Prevarskaya et al., 2018; Stelling et al., 2019). Recently, inspired by the potent anti-tumor effects induced by exogenous metals, therapeutic application of metallic elements has witnessed considerable attention in oncology toward iron, copper, manganese, zinc, calcium, and other metal elements (Andreini et al., 2008).

**SCHEME 1:**
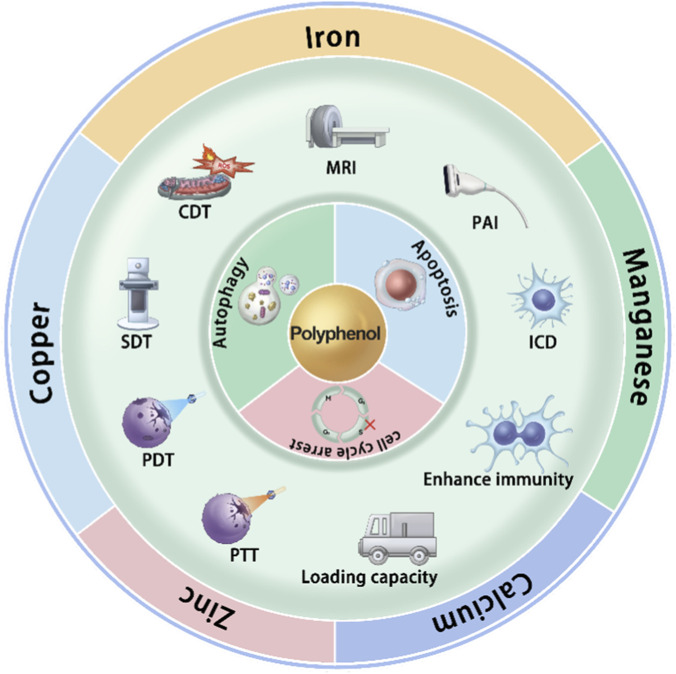
The main mechanisms of metal-polyphenol nanomedicines in anti-tumor therapy.

Polyphenols are natural organic compounds that belong to plant secondary metabolites and are widely found in vegetables, tea and other plant sources. They exhibit anti-inflammatory, anti-aging and anti-tumor activities, together with strong adhesion and UV-blocking properties (Li et al., 2022; Zhou et al., 2016). Owing to its potent tumor-suppressive efficacy, polyphenols have become a focus of anticancer research. Nevertheless, concentrations of polyphenols rarely reach 1 µM in plasma (Scalbert and Williamson, 2000). The poor bioavailability limits their therapeutic potential. Thanks to phenolic hydroxyls, polyphenols can assemble with various metal ions or molecules through covalent and non-covalent interactions to form nanomaterials, overcoming this drawback. More promisingly, metal-polyphenol systems are not limited to nanomedicines formed solely by coordination between a single metal and polyphenols. They can also incorporate other components such as drugs, as illustrated in Scheme 1, thereby exerting anticancer effects through multiple mechanisms including chemodynamic therapy, photothermal therapy, or immunotherapy.

## 2 Anti-tumor mechanisms of metal

**SCHEME 2:**
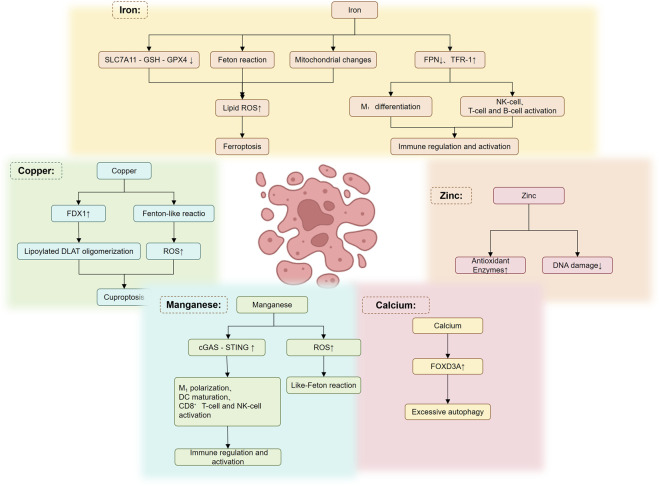
The main mechanisms of metal in anti-tumor therapy.

Iron, copper, manganese, calcium and zinc are essential metals. Beyond their basic metabolic roles, they weave a finely tuned anti-tumor network through ferroptosis, cuproptosis, cGAS-STING-pathway activation, mitochondrial Ca^2+^ overload, epigenetic reprogramming and reshaping of the immune microenvironment, which impinge on tumor initiation, progression and metastasis. The anti-cancer mechanisms of each metal are detailed in the following text and Scheme 2.

### 2.1 Anti-tumor mechanisms of iron

Dietary iron is Fe^3+^ mostly, but is reduced to Fe^2+^ by reductases in the duodenum before absorption. After uptake, Fe^2+^ is re-oxidized to Fe^3+^ and loaded onto serum transferrin (TF). The TF–Fe^3+^ complex is imported via membrane-bound transferrin receptor 1 (TFR1). Inside the endosome, Fe^3+^ is reduced back to Fe^2+^ by the six-transmembrane epithelial antigen of prostate 3 (STEAP3) and then released into the cytosol through divalent metal transporter 1 (DMT1) to join the labile iron pool (LIP). From the LIP, Fe^2+^ is distributed to multiple destinations where it participates in respiration, energy metabolism, DNA synthesis and repair and cell-cycle control (Kuang and Wang, 2019; Torti and Torti, 2013; Xl et al., 2022).

Iron restrains tumor growth chiefly by triggering ferroptosis and reshaping anti-tumor immunity. Ferroptosis is a distinct and non-apoptotic form of regulated cell death first described by Dixon et al., in 2012 as an iron-dependent process (Dixon et al., 2012). It is driven by unrestrained lipid peroxidation, clearly distinguished from apoptosis, necrosis or autophagy. Ferroptosis is typically accompanied by characteristic mitochondrial changes—mitochondrial shrinkage, increased membrane density, and reduced or absent cristae. The SLC7A11-GSH-GPX4 axis has emerged as the canonical intracellular pathway that restrains ferroptosis by preventing peroxidation of poly-unsaturated fatty acids (PUFAs) (Friedmann et al., 2014). In immune regulation, M_1_ polarisation is typically accompanied by downregulation of ferroportin (FPN) and upregulation of transferrin receptor-1 (TfR1) (Ho et al., 2022). Conversely, increased expression of TfR1 and lipocalin-2 biases macrophages toward the M_2_ phenotype (DeRosa and Leftin, 2021). Thus, macrophage polarization is tightly linked to the local iron milieu, while ample iron favors M_1_ differentiation, whereas iron scarcity promotes the M_2_ state. NK-cell activation also requires iron, because diminished TFR1 (CD71) activity precipitates iron deficiency and cripples NK cytotoxicity (Littwitz-Salomon et al., 2021). Recent studies show that TfR1 mutations reduce mature T-cell numbers (Vanoaica et al., 2014)and cause B-cell damage and dysfunction, leading to combined immunodeficiency (Jabara et al., 2016).

### 2.2 Anti-tumor mechanism of copper

Copper is an indispensable trace element in living systems that serves as a pivotal cofactor in redox signaling and electronic transfer reactions (Tang et al., 2024). Once its concentration exceeds the buffering capacity of homeostatic regulators, however, copper becomes cytotoxic (Chen et al., 2022). Therefore, this property can be exploited.

In 2022, copper-dependent cell death was formally recognized as a distinct form of regulated cell death called cuproptosis (Tsvetkov et al., 2022). Tumor cells acquire copper via the copper importer CTR1 (SLC31A1), which directly sets intracellular copper levels (Yu et al., 2019). Conversely, ATP-driven copper exporters ATP7A/B extrude Cu^+^ by coupling ATP hydrolysis to metal transfer, thereby fine-tuning copper homeostasis (Wang et al., 2023). Cuproptosis is initiated within mitochondria when matrix Cu^+^ rises, triggering oligomerization of lipoylated dihydrolipoamide transacetylase (DLAT) (Tsvetkov et al., 2022). This death modality is mechanistically linked to oxidative stress. Copper regulates transporters (CTR1 for import and ATP7A/B for export), then it amplifies cuproptosis via FDX1 driven activation of TCA cycle components under copper overload conditions (Vo et al., 2024).

### 2.3 Anti-tumor mechanism of manganese

Manganese is an essential trace element that participates in a broad spectrum of physiological processes, including immunity, hematopoiesis, endocrine function, and the modulation of oxidative stress (Chen et al., 2018; Horning et al., 2015). Manganese enhances immune function by increasing the sensitivity of the cGAS-STING pathway, stimulating dendritic cell (DC) maturation and macrophage M_1_ polarization (Hooy et al., 2020; Wang et al., 2018). Mn^2+^ not only enhances the ability of cGAS to catalyze the production of cGAMP at low dsDNA levels, but also increases the affinity between cGAMP and STING on the endoplasmic reticulum surface, leading to IRF3 phosphorylation and activation of the NF-κB pathway, thereby promoting the production of type I interferons (IFN-I) (Taguchi et al., 2021). On this basis, this pathway initiates via CD8^+^ T cells and NK cells (Lv et al., 2020; Song et al., 2021; Zhang et al., 2021). Secondly, Mn^2+^ can exert Fenton-like activity. In the presence of endogenous H_2_O_2_, it catalyzes the decomposition of H_2_O_2_ to generate highly toxic ·OH radicals, inducing oxidative damage in tumor cells and triggering apoptosis or necrosis (Lin et al., 2018).

### 2.4 Anti-tumor mechanism of calcium

Ca^2+^ is the most abundant metal ion in the human body and serves as a crucial second messenger in numerous cellular processes. It plays a key role in regulating cell proliferation, metabolism, migration, immunity, cell death, signal transduction, and gene expression (Giorgi et al., 2018; Zheng et al., 2023). Its function is particularly critical at all stages of tumor metastasis, where calcium-mediated signaling pathways regulate key oncogenic hallmarks, including angiogenesis, invasiveness, and migratory capacity (Bagur and Hajnóczky, 2017). In anti-tumor mechanisms, endoplasmic reticulum (ER) stress-induced calcium leakage has become a research hotspot. Specialized microdomains between mitochondria (MITO) and the ER, known as mitochondria-associated membranes (MAMs), act as signaling hubs for inter-organellar communication, regulating organelle function and metabolism. The inositol 1,4,5-trisphosphate receptor (IP3R), a protein localized at MAMs, is the primary ER calcium release channel (Wu et al., 2017). Notably, IP3R-mediated ER Ca^2+^ release results in mitochondrial calcium concentrations significantly higher than cytosolic free calcium, thereby triggering downstream signaling pathways and inducing apoptosis (Lampl et al., 2020). IP3R has emerged as a functional target for an increasing number of oncogenes and tumor suppressors that dynamically modulate its activity, thereby controlling Ca^2+^ flux from the ER into mitochondria (Akl and Bultynck, 2013; Bittremieux et al., 2016). Additionally, experimental studies have demonstrated that Ca^2+^-FOXO3A pathway-mediated excessive autophagy can induce osteosarcoma cell death (Kang et al., 2018).

### 2.5 Anti-tumor mechanism of zinc

As an essential trace element, zinc is indispensable for protein folding, conformational switching and activation during synthesis. It underpins a wide spectrum of biological processes, including nucleic-acid metabolism, DNA repair, antioxidant defense, transcription and controlled cell proliferation (Pan et al., 2017). Consequently, zinc has been implicated in cancer biology and is even regarded as a potential antineoplastic agent (Wong et al., 2015). Zinc is component of numerous enzymes that regulate the antioxidant defense system. Under the threat of oxidative damage, it stabilizes protein thiol groups, thereby preventing oxidative inactivation and lowering the reactivity of these thiols is an effective protective strategy (Emanuelli et al., 1998; Jarosz et al., 2017). Conversely, zinc deficiency heightens sensitivity to oxidative stress, provoking oxidative DNA lesions, such as single and double strand breaks and base oxidation that can initiate and propagate carcinogenesis (Al-Saran et al., 2016; Ho, 2004). Among the proteins involved in DNA synthesis and repair, more than 3,000 transcription factors and over 300 enzymes are associated with zinc (Yang et al., 2016). Moreover, the impact of zinc deficiency is not confined to cell cycle checkpoints and DNA repair, because many proteins or transcription factors that govern programmed cell death are zinc-finger or zinc-associated proteins (Ho, 2004). Zinc transporters exert profound effects on tumour progression and their expression profiles differ among neoplastic cell types, thereby modulating proliferation, invasion and metastasis (Cheng et al., 2017; Lopez et al., 2011). Which leads to a divergence whether zinc supplementation benefits or harms cancer patients.

## 3 Anti-tumor mechanisms of polyphenols

**SCHEME 3:**
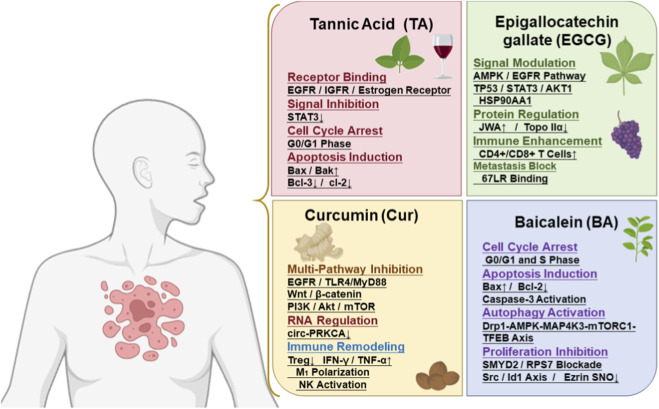
The main mechanisms of polyphenol in anti-tumor therapy.

Polyphenols are natural organic compounds classified as plant secondary metabolites, widely distributed in vegetables, tea, and other plant-derived foods. Their pleiotropic bioactivities, including antioxidant, anti-aging, and anti-tumor effects, have been extensively documented (Li et al., 2022; Zhou et al., 2016). Notably, the anti-tumor molecular mechanisms of different polyphenols exhibit considerable diversity. Herein, we briefly describe four representative anti-tumor mechanisms of polyphenols frequently implicated in metal-polyphenol complex systems, as outlined in this section and Scheme 3.

### 3.1 Anti-tumor mechanisms of Tanic (TA)

Tannic acid (TA) is a polyphenol commonly found, derived from plants, which is enriched in tea, red wine, nuts, legumes, vegetables, and tree bark (Nagesh et al., 2018). Due to its numerous hydroxyl functional groups, it can modulate oncogenic signals by directly binding to biomacromolecules (Darvin et al., 2017). It has been shown that tannic acid inhibits breast cancer cell viability by binding to cell surface growth factor receptors, including epidermal growth factor receptor (EGFR), insulin-like growth factor receptor (IGFR), and estrogen receptor (Booth et al., 2013). Additionally, tannic acid can downregulate signal transducer and activator of transcription 3 (STAT3) signaling and reduce cell viability (Darvin et al., 2015). In regulating the tumor cell cycle and apoptosis, tannic acid induces G1 arrest and apoptosis in breast cancer cells via bidirectional modulation of the STAT pathway (Li et al., 2024)and can promote TRAIL-mediated programmed cell death in embryonic cancer stem cells (Sp et al., 2020a). Notably, tannic acid can additionally target cancer stem cells by inhibiting NF-κB-mediated phenotypic transformation in breast cancer cells (Kim et al., 2019). Moreover, tannic acid demonstrates anti-tumor activity in non-small cell lung cancer by similarly inducing G0/G1 arrest and apoptotic pathways (Sp et al., 2020b). Moreover, it also triggers PARP and caspase-3 cleavage, upregulates pro-apoptotic Bax/Bak proteins, and downregulates anti-apoptotic Bcl-2 to enhance apoptosis (Li et al., 2024).

### 3.2 Anti-tumor mechanisms of epigallocatechin-3-gallate (EGCG)

EGCG is one of the most important active components in green tea, capable of scavenging free radicals and reducing oxidative damage, thereby exerting beneficial effects in tumor prevention and treatment (Aggarwal et al., 2022). Studies have demonstrated that EGCG exerts anti-tumor effects against NSCLC via multiple mechanisms, including modulation of AMPK and EGFR signaling pathways, upregulation of JWA protein and downregulation of topoisomerase IIα (topo IIα) (Chen et al., 2020; Li et al., 2015; Minnelli et al., 2021). Furthermore, EGCG can bind to the metastasis-associated 67 kDa laminin receptor (67LR), leading to inhibiting malignant tumor metastasis (Tachibana et al., 2004; Umeda et al., 2008). Furthermore, the combination of EGCG with chemotherapy improves therapeutic outcomes while enhancing immune function through the upregulation of CD4^+^/CD8^+^ T cells (Laurie et al., 2005). Recently, based on the intersection analysis of EGCG potential targets and NSCLC disease-related genes, studies that integrated network pharmacology, bioinformatics, and molecular docking technologies have successfully identified a core regulatory network comprising TP53, STAT3, AKT1, IL-6, HSP90AA1, and JUN. Molecular docking results revealed that EGCG exhibits high-affinity binding to all targets within this network, with the binding energy to TP53 and HSP90AA1 being particularly pronounced (Yang et al., 2025). Future experimental validation of these pathways is anticipated.

### 3.3 Anti-tumor mechanisms of curcumin (Cur)

Curcumin, a natural polyphenol from turmeric, has antioxidant, anti-inflammatory, and anti-tumor effects (Li et al., 2018; Wang et al., 2017; Xu and Zhu, 2017). It combats various cancers by modulating multiple molecular targets and signaling pathways (Rodrigues et al., 2019). In non-small cell lung cancer (NSCLC), curcumin suppresses malignancy by regulating EGFR and TLR4/MyD88 pathways (Zhang et al., 2019). Additionally, it inhibits Wnt/β-catenin (Pan et al., 2020) and PI3K/Akt/mTOR pathways (Liu et al., 2018). It also stunts NSCLC growth by downregulating circ-PRKCA, which sponges miR-384 to control ITGB1 expression (Xu et al., 2021). In gastric cancer, curcumin inhibits proliferation by inducing apoptosis in tumor cells, activating immune cells to secrete large amounts of cytokines and downregulating signaling pathways, such as the DEC1, HIF-1α, VEGF and STAT3 (Wang et al., 2017). In retinoblastoma, curcumin exerts anti-tumor effects by modulating the JNK and p38 MAPK pathways (Yu et al., 2016). Curcumin can also inhibit the metastasis of Wilms’ tumor by suppressing RECK methylation (Jia et al., 2019). In immunomodulation, curcumin excels at inhibiting Tregs activity through three main mechanisms. It alters the immune system’s response to Th1 within the tumor microenvironment (TME), enhances the release of IFN-γ and TNF-α and promotes the proliferation of anti-tumor immune cells such as CD8^+^ T cells and NK cells. Additionally, curcumin promotes the polarization of macrophages into M_1_ macrophages and modulates the interaction between TAM, M_1_ macrophages and NK cells, thereby enhancing the anti-tumor activity of NK cells (Fu et al., 2021; Jiang et al., 2022; Shafabakhsh et al., 2019).

### 3.4 Anti-tumor mechanisms of baicalein (BA)

Baicalein (BA), belonging to flavone and extracted from Scutellaria baicalensis, is chemically defined as 5,6,7-trihydroxyflavone and possesses pronounced anti-inflammatory, antimicrobial and immunomodulatory effects (Liao et al., 2021). Baicalein is able to arrest tumor cell cycle. Experiments show that baicalein lowers cyclin A expression and reduces cyclin D1 to impose G0/G1 (Gao et al., 2011) as well as S-phase blocks (Lee et al., 2005). In terms of apoptosis, baicalein shifts this balance toward death by up-regulating Bax, down-regulating Bcl-2 and activating caspase-3 (Chandrashekar et al., 2022). In A549 and H1299 lung cancer cells, baicalein appears to trigger both apoptosis and autophagic cell death by promoting Drp1-mediated mitochondrial fission and resulting in AMPK activation (Deng et al., 2020). As for autophagy, baicalein is the first reported small-molecule ligand of MAP4K3. Hence, baicalein suppresses non-small-cell lung cancer through the MAP4K3-mTORC1-TFEB axis (Li et al., 2021). Moreover, baicalein dose-dependently blocks the SMYD2/RPS7 signaling cascade, inhibiting A549 cell proliferation and markedly reducing both invasive capacity and clonogenic survival (Gu et al., 2022). Lastly, baicalein curbs migration and metastasis by targeting the Src/Id1 axis (Zhao et al., 2019) and decreasing S-nitrosylation (SNO) of Ezrin (Zhang et al., 2020).

## 4 Metal-polyphenol nanomedicines

Metal-polyphenol self-assembles into carbon dots, nanonetworks, core-shell particles and more, which not only exert direct anti-tumor activity but also shield the nanocarriers from premature degradation. Like other nanoplatforms, metal-polyphenol nanomedicines can efficiently load drugs. Owing to their straightforward preparation and outstanding photothermal performance, metal-polyphenol nanostructures have become a research frontier in oncotherapy. These systems can independently manifest the antineoplastic actions derived from both the metal ions and the polyphenols, and they can also interact synergistically to potentiate therapeutic efficacy. Here, we summarize the latest anti-tumor strategies and mechanisms reported for metal-polyphenol nanomedicines in recent years, with details provided in Table 1.

**TABLE 1:** Metal-polyphenol coordination nanomedicines for malignant-tumor therapy.

Metal	Polyphenol	Additional metals	Loading	Material design	Main advantages	References
Fe						
​	APG	​	​	β-CD/PEG-stabilized Fe-APG NPs	​	Chen et al. (2024b)
	Cur	​	​	Ultrasound molecular probe FCIPL	Quadruple-modal imaging; ultrasound cavitation for deep penetration	Dong et al. (2025)
	EA	​	​	pH/H_2_O_2_ dual-responsive Fe-EA framework	Tumor specific dissociation	Zhao et al. (2018)
	EA	​	​	PDA core + Fe-EA network + PEG shell	PTT synergizes with ferroptosis; tyrosinase inhibition	Wang et al. (2025)
	EGCG	​	​	HA microneedles delivering EFP nanocapsules	Multimodal synergy; immunomodulation; CD44 targeting	Wang et al. (2024a)
	EGCG	​	​	Low-temperature PTT-enhanced CDT	EGCG downregulates HSP90 for eliminating thermal tolerance	Yu et al. (2021)
	EGCG	Ce	​	EGCG-Fe coordination encapsulating Ce6	Ferroptosis; PDT; CD44 targeting	Liang et al. (2022)
	EGCG	Ce	​	Core-shell MOF: Ce-aMOF core + Fe-EGCG shell	Dual-enzyme activity depletes antioxidant system	Zhang et al. (2023)
	EGCG	​	DOX	Mesoporous silica loaded with DOX, Fe-EGCG shell	pH-responsive release; multimodal synergy	Wang et al. (2024b)
	EGCG	​	DOX	Pluronic F127-stabilized Fe-EGCG framework	Trimodal synergy; high drug loading	Shi et al. (2023)
	EGCG	​	DOX/BPTES	Fe-EGCG framework co-loading GLS1 inhibitor	Metabolic intervention; chemotherapy synergy	Dai et al. (2024)
	EGCG	​	Pt-OH	EGCG-platinum (IV) complex, PEG-b-PPOH modified	High drug loading; cascade reaction generating H_2_O_2_	Ren et al. (2020)
	GA/Art	​	​	MIL-100/artemisinin core + Fe-GA/HA shell	PTT; chemotherapy; ferroptosis	Zhang et al. (2025)
	GA	Mn	​	Fe-GA nanodots embedded in hollow MnO_2_, pH-responsive PEG shielding	pH/GSH-responsive release; hollow structure enhances deep penetration	Duan et al. (2023)
	GA	Mn	CAI	MnO_2_@GA-Fe@CAI, Fe^2+^/Fe^3+^/Mn^2+^ synergy	Multi-metal ion catalysis; TME remodeling	Chen et al. (2024a)
	GA	​	PTX	Loaded with paclitaxel + Fe-GA shell	Loaded with drug; mitochondrial ATP synthesis inhibition	Xu et al. (2023)
	GA	Ca	Pt-SA	Hollow GA-Fe@CaCO_3_ loaded with cisplatin	pH/GSH-responsive release	Han et al. (2022)
	GA	Ca	Pt/DOX	Hollow GA-Fe@CaCO_3_ loaded with cisplatin/doxorubicin	pH/GSH-responsive release	Dong et al. (2020)
​	PA	​	​	Fe crosslinked gelatin-protocatechuic acid hydrogel	ICD induction; TGF-β inhibition	Guo et al. (2025)
	PGG	​	Haase	Hyaluronidase-embedded PGG-Fe network	Self-degradation; CD44 targeting; immune microenvironment remodeling	Sun et al. (2024)
	Art/Ru	Zn	​	ZIF-8+Ru-Fe network	GLUT targeting; CDT	Zhang et al. (2024a)
​	Ru	Zn	PTX	ZIF-8 loaded drug + Ru-Fe network	GLUT targeting; CDT; Chemotherapy; Cell cycle arrest	Lu et al. (2024)
	SAB	​	​	FeSH NPs	PTT-PDT dual-modal imaging	Meng et al. (2024)
	Shikonin	​	​	Fe (III)-shikonin + PEG-cRGD	Ferroptosis; apoptosis synergy; cRGD targeting; MRI imaging	Feng et al. (2022a)
	Shikonin	​	SRF/GO_X_	Fe (III)-shikonin + SRF + GO_X_ anchoring	Quadruple strike; cascade amplification	Feng et al. (2022b)
	TA	​	​	Bacterial outer membrane vesicles + Fe-TA coating	ICD induction; cGAS-STING activation; M_2_→M_1_ repolarization	Nie et al. (2024)
	TA	​	​	Magnetic nanorobot Fe_3_O_4_-TA-Fe	Magnetic-driven navigation; cascade catalysis	Li et al. (2025a)
	TA	Mn	​	Bacterial outer membrane vesicles + Fe-TA coating (with Mn)	ICD induction; cGAS-STING activation; M_2_→M_1_ repolarization	Sun et al. (2023)
	TA	Au	​	Gold nanomaterial core + Fe-TA shell	Cascade catalysis; active targeting	Peng et al. (2022)
	TA	Au	​	Gold nanomaterial core + Fe-TA shell	Cascade catalysis; active targeting; PTT	Leng et al. (2025)
	TA	Er/Ce/Tm	​	Upconversion nanoparticles + Fe-TA	Theranostics; MRI/NIR-II imaging guidance	Qu et al. (2025)
	TA	​	HBP1	—	CDT-Chemotherapy synergy	Yang et al. (2023)
	TA	Cu	DOX	CuS-MPDA core + Fe-TA matrix	PTT-chemotherapy synergy	Wang et al. (2024c)
	TA	​	DACH-Pt (II)	Pt-TA nanocomplex	ICD induction; high drug loading	Xiang et al. (2022)
	TA	Mn	R848	Mesoporous silica + R848+Fe/Mn-TA coating	Local implantation; immune microenvironment remodeling	Li et al. (2025b)
	TA	​	SRF/NAPP	—	CDT; PDT; Chemotherapy	Zhou et al. (2022)
	TA	Cu	GO_X_	Nanomaterial core + Fe-TA shell (Cu/GO_X_ version)	Cascade catalysis; active targeting	Li et al. (2025a)
	TA	Zn	BAY-876	HA-ZnO_2_-ZIF-8 cascade structure	Ferroptosis-disulfidptosis synergy; cGAS-STING activation	Su et al. (2024)
	TA	​	SsPPE	β-polyphosphate core + Fe-TA shell	Disulfidptosis-ferroptosis-CDT synergy	Dai et al. (2023)
	TA	​	TPZ/GO_X_	—	CDT-Chemotherapy-Starvation therapy	Guo et al. (2020)
	TA	​	BLM/ML210	Prussian blue loaded with ML210+TA-BLM-Fe	Ferroptosis- Chemotherapy-Apoptosis synergy	Zhou et al. (2021)
Cu						
​	EGCG	​	​	Cu-EGCG coordination network	Cyclic catalysis; antibacterial-antitumor dual function	Chen et al. (2024c)
	EGCG	​	Pt	EGCG-Cu-Pt nanomedicine	Platinum drug co-loading; TME-responsive release	An et al. (2024)
	TA	​	​	TA-TCNQ-Cu^2+^ ternary complex	Cuproptosis-ferroptosis synergy; PTT-enhanced cuproptosis; mitochondrial targeting	Liang et al. (2025)
	TA	​	​	Cu-TA network + liposome-encapsulated STF-31	Glycolysis inhibition-cuproptosis-ferroptosis synergy; immune microenvironment remodeling	Zhang et al. (2024b)
Mn						
​	EGCG	​	SRF	BSA-stabilized Mn-EGCG encapsulating sorafenib	cGAS-STING activation; excellent biocompatibility	Cai et al. (2025)
	GA	​	​	Metal-phenol carbon dots by hydrothermal carbonization	Carbon dot innovative application; DAMPs release	Wu et al. (2022)
	PDA	Zn	​	ZIF-8/MnCO@PDA core-shell structure	CDT-PTT-immunotherapy-gas therapy quadruple synergy; CO gas therapy	Zhang et al. (2024c)
	TA	​	​	IMT@H polyphenol network loaded with IR780	Prophylactic treatment potential; precise mitochondrial damage	Liu et al. (2025)

Comment: APG: apigenin; Art: Artemisinin; Cur: Curcumin; EA: ellagic acid; EGCG: epigallocatechin gallate; GA: gallic acid; PGG: 1,2,3,4,6-Penta-O-galloyl-β-D-glucose; Ru: Rutin; SAB: Salvianolic Acid B; TA: tannic acid; PDA: Polydopamine. 5-FU: 5-Fluorouracil; BAY-876: Glucose transporter inhibitor; BLM: bleomycin; BPTES: Bis-2-(5-phenylacetamido-1, 3,4-thiadiazol-2-yl)ethyl sulfide; CAI: carbonic anhydrase inhibitor; DACH-Pt (II): 1,2-Diaminocyclohexane-Pt (II); DOX: doxorubicin; GO_X_: glucose oxidase inhibitor; Haase: Hyaluronidase; HBP1: transcription factor; ML210: GPX4 inhibitor; NAPP: resiquimod; Pt: Cisplatin; Pt (IV)-SA: phenolic platinum prodrug; Pt-OH: phenolic platinum (IV) prodrug; PTX: paclitaxel; R848: Resiquimod; SRF: sorafenib; ssPPE: a disulfide-linked β-Polyphosphoester; TPZ: Tirapazamine prodrug. CDT: chemodynamic therapy; ICD: immunogenic cell death; MRI: magnetic resonance imaging; PAI: photoacoustic imaging; PDT: photodynamic therapy; PTT: photothermal therapy; SDT: sonodynamic therapy.

### 4.1 Iron-polyphenol nanomedicines

Iron is an essential element that exists in two forms that is organic heme iron and inorganic non-heme iron. The former is embedded in hemoglobin and myoglobin, mediating blood oxygen transport and muscle oxygen storage, respectively (Abbaspour et al., 2014), whereas the latter is sequestered by proteins such as ferritin, hemosiderin and neuromelanin (Seo et al., 2008). Inside cells, iron homeostasis is tightly controlled by iron-regulatory proteins (IRPs) and iron-responsive elements (IREs) at the transcriptional and translational levels, ensuring a precise balance among uptake, storage, export and distribution. When iron overload occurs, free Fe^2+^ catalyzes a burst of radicals via the Fenton reaction, provoking oxidative stress that can trigger decisive cell-death or survival signaling pathways (Reinert et al., 2022). Among the various metal-polyphenol nano-platforms developed for malignancies, iron-polyphenol systems have attracted the greatest attention. The following sections provide a detailed overview of the most frequently reported iron-polyphenol assemblies, focusing on their material composition and anti-tumor mechanisms.

#### 4.1.1 Iron-epigallocatechin gallate (EGCG)

Fe-EGCG nanomedicines form metal-polyphenol coordination networks that combine ferroptosis, photothermal therapy, photodynamic therapy, chemodynamic therapy, and chemotherapy into a single platform (Dai et al., 2024; Liang et al., 2022; Ren et al., 2020; Shi et al., 2023; Wang W. et al., 2024; Wang S. et al., 2024; Yu et al., 2021; Zhang et al., 2023). EGCG depletes glutathione and regulates multiple targets, while iron ions drive Fenton reactions to amplify oxidative damage (Dai et al., 2024; Liang et al., 2022; Ren et al., 2020; Shi et al., 2023; Wang W. et al., 2024; Wang S. et al., 2024; Yu et al., 2021; Zhang et al., 2023). Researchers have developed microneedle patches and core-shell structures for tumor microenvironment-responsive drug release and immune remodeling (Wang W. et al., 2024; Zhang et al., 2023). These systems offer multimodal synergy, high drug loading, and simple preparation (Dai et al., 2024; Ren et al., 2020; Shi et al., 2023; Wang S. et al., 2024).

Among various Fe-EGCG nanomedicine systems, the multifunctional microneedle patch (EFP@MNs) developed by Pan et al. stands out for its distinctive design. This patch can be directly applied to the tumor skin surface, enabling minimally invasive local drug delivery that significantly reduces patient discomfort and systemic side effects. Upon insertion into the tumor, the released EFP nanocapsules trigger lipophagy-driven metabolic disturbances, promoting lipid peroxidation (LPO) and ferroptosis. When combined with PTT, the platform further remodels the tumor immune microenvironment by repolarizing tumor-associated macrophages toward the pro-inflammatory M_1_ phenotype and promoting dendritic cell maturation (Wang W. et al., 2024). For superficial tumors, this patch design offers more direct therapeutic effects and lower systemic toxicity compared to other delivery systems. Another notable design is the MEF core-shell nanoparticles constructed by Wang et al. This system utilizes cerium-based amorphous metal-organic frameworks (Ce-aMOFs) as the core, coated with an outer Fe-EGCG shell to form a stable multilayered structure. In the tumor microenvironment, MEF simultaneously releases Fe^3+^ and EGCG while exposing the internal Ce-aMOF. On one hand, EGCG and Fe^3+^ synergistically deplete GSH. On the other hand, Ce-aMOF exhibits dual enzyme-like activities, including SOD-like activity and phosphatase-like activity. This multi-pronged system depletion strategy, coupled with its structural stability, ultimately achieves marked amplification of ferroptosis for synergistic therapy (Zhang et al., 2023). Additionally, Jiao et al. achieved a breakthrough in metabolic intervention. They similarly employed Fe-EGCG coordination to construct a novel metal-phenolic multifunctional nanomedicine, Fe-DBEF, stabilized by Pluronic F127. The innovative aspect of this framework is the co-delivery of the glutaminase 1 (GLS1) inhibitor BPTES and doxorubicin (DOX). BPTES blocks glutamine metabolism, thereby cutting off the precursor supply for GSH synthesis and weakening tumor cell antioxidant defenses at the metabolic source. Combined with CDT and chemotherapy, this approach achieves a deeper level of synergistic antitumor efficacy (Dai et al., 2024).

However, several challenges hinder clinical translation. Iron overload and high-dose EGCG may cause systemic oxidative stress, liver toxicity, and immune overactivation. Notably, no long-term safety data currently exists (Dai et al., 2024; Liang et al., 2022; Ren et al., 2020; Shi et al., 2023; Wang W. et al., 2024; Wang S. et al., 2024; Yu et al., 2021; Zhang et al., 2023). The nanomedicines also show limited biological stability, as sensitivity to GSH and pH in blood circulation can trigger premature disassembly. Tumor microenvironment heterogeneity further reduces treatment consistency (Liang et al., 2022; Yu et al., 2021). Scaling up from milligram laboratory batches to kilogram industrial production presents quality control, purification, and manufacturing challenges (Dai et al., 2024; Ren et al., 2020; Shi et al., 2023; Wang S. et al., 2024). Regulatory hurdles include unclear approval pathways for novel nanodrugs, lack of specific evaluation guidelines, and gaps between animal models and human patients. Complex pharmacokinetics and high production costs add additional barriers (Dai et al., 2024; Liang et al., 2022; Ren et al., 2020; Shi et al., 2023; Wang W. et al., 2024; Wang S. et al., 2024; Yu et al., 2021; Zhang et al., 2023).

#### 4.1.2 Iron-gallic acid (GA)

Fe-GA self-assembled nanomaterials primarily rely on chemodynamic therapy (CDT) for tumor treatment, with recent advances demonstrating sophisticated material designs including core-shell architectures (MIL-100/artemisinin core with Fe^3+^-GA/HA shell (Zhang et al., 2025), MnO_2_@GA-Fe@CAI (Chen W. et al., 2024), and hollow GA-Fe@CaCO_3_ (Dong et al., 2020; Han et al., 2022), pH-responsive coatings (benzoic-imine bonded mPEG/PEI (Duan et al., 2023), and multi-metal ion strategies (Fe^2+^/Fe^3+^/Mn^2+^) to enhance CDT efficacy (Chen W. et al., 2024; Dong et al., 2020; Duan et al., 2023; Han et al., 2022; Xu et al., 2023; Zhang et al., 2025). These systems achieve synergistic anti-tumor effects through multiple mechanisms, such as continuous •OH generation via Fenton/Fenton-like reactions (Duan et al., 2023; Zhang et al., 2025), glutathione depletion and H_2_O_2_ self-supply by MnO_2_ components (Chen W. et al., 2024; Duan et al., 2023), tumor microenvironment remodeling through pH reduction and metabolic inhibition (Chen W. et al., 2024), and integration of photothermal therapy, chemotherapy, and ferroptosis induction (Dong et al., 2020; Han et al., 2022; Xu et al., 2023; Zhang et al., 2025). Notably, the Fe^3+^-GA network enables integrated diagnosis and treatment as photoacoustic and T_1_-weighted MRI contrast agents (Zhang et al., 2025), while stimuli-responsive designs ensure tumor-specific drug release and minimize off-target effects (Dong et al., 2020; Duan et al., 2023; Han et al., 2022).

Among Fe-GA nanomedicines, the MAGFH platform constructed by Xu et al. features a particularly exquisite design. This system employs MIL-100 loaded with artemisinin (Art) as the core, enveloped by an outer Fe-GA network and hyaluronic acid (HA) targeting shell. Upon cellular uptake, MAGFH undergoes Fenton reactions, while Fe^2+^ triggers the burst release of •O_2_^−^/•C radicals from artemisinin, synergistically inducing apoptosis and ferroptosis under 808 nm laser irradiation. Moreover, the Fe-GA network functions as both photoacoustic imaging (PAI) and T_1_-weighted MRI contrast agents, achieving integrated diagnosis and treatment (Zhang et al., 2025). The distinctive feature of this design lies in that the outer metal-polyphenol network and inner drug core can function independently yet cooperatively interact with each other. Li et al., in contrast, explored a multi-metal synergistic strategy. They embedded Fe-GA nanodots within hollow manganese dioxide nanoparticles (HMDN), sealed with polyethyleneimine (PEI) and further grafted with methoxy-polyethylene glycol (mPEG), constructing the GA-Fe@HMDN-PEI-PEG system through pH-sensitive benzoic-imine bonds. The mPEG layer masks PEI to reduce non-specific uptake by normal cells, prolong blood circulation, and enhance tumor accumulation. In the acidic tumor microenvironment, PEG detachment exposes positively charged PEI, facilitating cancer cell internalization. Subsequently, HMDN consumes GSH and releases Mn^2+^, while Fe-GA nanodots provide Fe^2+^/Fe^3+^; GA reduces high-valence metals to achieve Mn^2+^/Fe^2+^ regeneration, continuously driving Fenton-like reactions to amplify •OH production (Duan et al., 2023). Although these two systems do not directly achieve multi-polyphenol-multi-metal coordination, they provide important directions for constructing more complex multi-polyphenol-multi-metal nanomedicines in the future. The remaining Fe-GA systems are relatively conventional in therapeutic strategy and structural design, showing no significant breakthrough features.

However, critical limitations of Fe-GA systems remain. Increasing structural complexity challenges batch reproducibility and scale-up manufacturing (Chen W. et al., 2024; Dong et al., 2020; Duan et al., 2023; Han et al., 2022; Xu et al., 2023; Zhang et al., 2025). In addition, all studies lack long-term toxicity data and validated biomarkers for patient stratification (Chen W. et al., 2024; Dong et al., 2020; Duan et al., 2023; Han et al., 2022; Xu et al., 2023; Zhang et al., 2025).

#### 4.1.3 Iron-tannic acid (TA)

Due to cost-effective coordination chemistry and versatile functionality (Dai et al., 2023; Guo et al., 2020; Leng et al., 2025; Li D. et al., 2025; Nie et al., 2024; Peng et al., 2022; Qu et al., 2025; Su et al., 2024; Sun et al., 2023; Wang Q. et al., 2024; Xiang et al., 2022; Yang et al., 2023; Zhou et al., 2021; Zhou et al., 2022), tannic acid is the most widely applied polyphenol in metal-polyphenol nano-systems, where co-loading metabolic inhibitors and chemotherapeutic drugs to synergistically activate CDT, PDT, and immunotherapy has become the mainstream design paradigm. These systems integrate multiple therapeutic modalities including chemodynamic therapy, photothermal therapy, photodynamic therapy, immunotherapy, and chemotherapy through sophisticated material designs such as bacterial outer-membrane vesicle coatings (Nie et al., 2024; Sun et al., 2023), gold nanorod cores (Leng et al., 2025; Peng et al., 2022), and cascade-structured nanorobots (Li D. et al., 2025; Su et al., 2024).

While a more forward-looking strategy lies in deeply integrating metal-polyphenol networks with bioactive molecules, achieving a transition from simple drug carriers to bio-functional regulation platforms. In terms of natural immune carriers, Xie’s team assembled a Fe-TA shell on the surface of bacterial outer-membrane vesicles (OMVs), which not only attenuates the systemic toxicity of naked OMVs but also enables intelligent disassembly in the tumor microenvironment. The released Fe^3+^ drives Fenton reactions to promote immunogenic cell death (ICD), generating tumor antigens that fuse with OMVs to form an inside vaccine; meanwhile, TA reduces Fe^3+^ to Fe^2+^, accelerating the Fenton reaction and achieving synergy between chemodynamic therapy and immunotherapy (Nie et al., 2024). More significantly, Zhang’s team introduced Mn^2+^ on this basis, constructing the dual-metal-polyphenol coordinated TA-Fe/Mn-OVA@MB system. This material simultaneously activates the Mn^2+^-mediated cGAS-STING pathway during local PTT, promoting dendritic cell maturation and antigen presentation, thereby activating cytotoxic T lymphocytes in peripheral lymphoid organs and inducing memory T-cell differentiation, offering a novel strategy for long-lasting anti-tumor immunity (Sun et al., 2023). In the realm of gene regulation and ferroptosis sensitization, research conducted by Zhang’s team demonstrated a distinct approach. This team leveraged the property of transcription factor HBP1 to downregulate UHRF1 and upregulate the CDO1 axis, thereby weakening the antioxidant capacity of tumor cells, and assembled it with the Fe-TA network into MPN-HBP1 nanoparticles (Yang et al., 2023). This system not only retains the classic chemodynamic function of Fe-TA but also actively lowers the ferroptosis threshold of tumor cells through HBP1-mediated metabolic reprogramming, thereby synergistically amplifying ferroptosis effects and achieving a strategic upgrade from passive drug delivery to active metabolic sensitization.

However, these systems face risks of acute metal toxicity from Fe^2+^/Cu^2+^/Mn^2+^ overload, potential systemic inflammation from bacterial vesicle components (Nie et al., 2024; Sun et al., 2023), and risks of immune hyperactivation when combined with immune checkpoint blockade (Dai et al., 2023; Xiang et al., 2022). Biological stability challenges include premature disassembly in circulation, metal ion leakage catalyzing harmful ROS in normal tissues, and enzymatic degradation of TA (Dai et al., 2023; Guo et al., 2020; Leng et al., 2025; Li D. et al., 2025; Nie et al., 2024; Peng et al., 2022; Qu et al., 2025; Su et al., 2024; Sun et al., 2023; Wang Q. et al., 2024; Xiang et al., 2022; Yang et al., 2023; Zhou et al., 2021; Zhou et al., 2022). Clinical translation faces substantial obstacles including undefined regulatory pathways for bio-inorganic hybrid systems, lack of validated biomarkers for patient stratification and limited penetration depth of photothermal and photodynamic components in deep tumors (Leng et al., 2025; Wang Q. et al., 2024). Future efforts must prioritize simplified designs with comprehensive immunotoxicity assessment and development of predictive biomarkers to advance these promising toward clinical application.

#### 4.1.4 Iron-shikonin

Iron-shikonin nanomedicines combine Fe(III)-shikonin coordination networks with targeted delivery and multi-modal therapy for cancer treatment (Feng et al., 2022a; Feng et al., 2022b). These systems integrate ferroptosis induction, apoptosis, and starvation therapy through GSH-responsive drug release and cascade reactions (Feng et al., 2022a; Feng et al., 2022b). Key features include αvβ_3_ integrin targeting via cRGD peptides, T_1_ MRI imaging capability, and triple-attack mechanisms involving Fenton reactions, glucose oxidase-catalyzed H_2_O_2_ generation, and sorafenib-blocked Xc^−^ system (Feng et al., 2022a; Feng et al., 2022b). Compared with other metal-polyphenol systems, Fe-Shikonin nanomedicines appear relatively conventional in terms of structural innovation and therapeutic diversity, but the inherent pharmacological activity of shikonin still endows this system with considerable development potential. Nevertheless, critical challenges remain in acute metal toxicity, enzymatic stability, regulatory classification, and clinical translation (Feng et al., 2022a; Feng et al., 2022b).

#### 4.1.5 Iron-other polyphenol

Except for EGCG, GA, TA, and shikonin, diverse polyphenols including apigenin, curcumin, ellagic acid, protocatechuic acid, pentagalloyl glucose, salvianolic acid B, and rutin have been coordinated with iron for cancer therapy, each leveraging distinctive iron-binding modes to trigger ferroptosis and/or other therapeutic pathways (Chen R. et al., 2024; Dong et al., 2025; Guo et al., 2025; Lu et al., 2024; Meng et al., 2024; Sun et al., 2024; Wang et al., 2025; Zhang LR. et al., 2024; Zhao et al., 2018). These systems are using cyclodextrin (Chen R. et al., 2024), pH/H_2_O_2_ dual-responsive frameworks (Zhao et al., 2018), polydopamine core-shell structures (Wang et al., 2025), hydrogel networks (Guo et al., 2025), enzyme-embedded metal-phenolic coatings (Sun et al., 2024), and MOF-based carriers (Lu et al., 2024; Zhang LR. et al., 2024). Key advantages encompass multi-modal imaging capabilities (Dong et al., 2025), deep tumor penetration via ultrasound cavitation (Dong et al., 2025), immune microenvironment remodeling through ICD induction and PD-L1 upregulation (Guo et al., 2025; Meng et al., 2024; Sun et al., 2024), metabolic intervention such as tyrosinase inhibition (Wang et al., 2025), and active targeting via GLUT or CD44 receptors (Lu et al., 2024; Sun et al., 2024; Zhang LR. et al., 2024).

The most distinctive designs are the following two systems. Zhang et al. ingeniously introduced a dual-metal and dual-polyphenol strategy into the nano-system, although only Fe-Ru undergoes direct coordination. This system uses ZIF-8 as the core loaded with artemisinin (Art), coated with an outer Fe-Ru network to form Ru-Fe@Art/ZIF. In the acidic tumor microenvironment, the released Art and Fe^2+^ from the disintegrated construct synergistically generate radical-dependent cell cycle arrest, significantly enhancing tumor killing efficacy, while rutin, as a functional polyphenol, plays multiple roles. On one hand, it mediates active targeting of tumor cells via GLUT receptors, and on the other hand, reduces Fe^3+^ to Fe^2+^ to accelerate the Fenton reaction (Zhang LR. et al., 2024). To address the bottleneck of dense and rigid tumor extracellular matrix (TECM) that severely hinders drug delivery, the research team constructed an integrated ZIF-8 nano-assembly, Ru/CCDs-PTX@ZIF. This system combines synergistic catalysis with chemotherapy. Through GLUT receptor-mediated endocytosis, the loaded paclitaxel (PTX) exerts tumor toxicity and inhibits cell migration. After entering the tumor, the dissociated Fe-Ru carbon dots exhibit peroxidase (POD)-like activity, generating abundant •OH to impose radical-dependent cell cycle arrest, thereby overcoming the matrix barrier to halt tumor progression (Lu et al., 2024). Notably, although both systems introduce ZIF-8 as a carrier, the potential role of zinc in tumor therapy has not been thoroughly explored. If the functionality of ZIF-8 can be further expanded beyond its role as a mere carrier, these systems would possess greater therapeutic appeal and clinical application value.

However, overall biological stability challenges include acute metal toxicity, photosensitizer-related phototoxicity, and risks of immune hyperactivation when combined with checkpoint blockade (Chen R. et al., 2024; Dong et al., 2025; Guo et al., 2025; Lu et al., 2024; Meng et al., 2024; Sun et al., 2024; Wang et al., 2025; Zhang LR. et al., 2024; Zhao et al., 2018). Clinical translation faces substantial obstacles including undefined regulatory pathways for complex bio-inorganic systems, lack of validated biomarkers for patient stratification, heterogeneity in receptor expression, and dependence on specialized equipment such as ultrasound devices (Chen R. et al., 2024; Dong et al., 2025; Guo et al., 2025; Lu et al., 2024; Meng et al., 2024; Sun et al., 2024; Wang et al., 2025; Zhang LR. et al., 2024; Zhao et al., 2018).

### 4.2 Copper-polyphenol nanomedicines

Copper, as the second most abundant trace element in the human body, has emerged as a promising therapeutic agent in cancer nanomedicine when coordinated with polyphenols, leveraging its unique redox properties to trigger cuproptosis and amplify oxidative stress (An et al., 2024; Chen Y. et al., 2024; Kim et al., 2008; Liang et al., 2025; Tan et al., 2014; Zhang Y. et al., 2024). Over thirty copper-polyphenol nanomaterials have been reported, yet fewer than ten have been explored for tumor applications. Researchers have explored multi-modal therapies including chemodynamic therapy, photothermal therapy, photodynamic therapy, cuproptosis, ferroptosis, and immunotherapy (An et al., 2024; Chen Y. et al., 2024; Liang et al., 2025; Zhang Y. et al., 2024). These systems employ sophisticated designs such as Cu-EGCG coordination networks integrating antibacterial activity (Chen Y. et al., 2024), EGCG-Cu-Pt nanoparticles for cisplatin-copper synergistic release (An et al., 2024), TA-TCNQ-Cu complexes with mitochondrial targeting and copper-efflux pump inhibition (Liang et al., 2025), and Cu-TA/liposome bilayers encapsulating glycolytic inhibitors for metabolic intervention (Zhang Y. et al., 2024). Key advantages include dual metal-death mechanisms that is cuproptosis-ferroptosis synergy, comprehensive metabolic suppression through glycolysis inhibition and GSH synthesis blockade, immune microenvironment remodeling via immunogenic cell death, and antibacterial-anti-tumor dual functionality (An et al., 2024; Chen Y. et al., 2024; Liang et al., 2025; Zhang Y. et al., 2024). However, compared with the highly diversified designs of iron-polyphenol systems, copper-polyphenol nanomedicines appear relatively homogeneous and conventional in terms of structural innovation and therapeutic strategies. Existing systems largely follow the classical coordination-release model, primarily relying on the redox properties of copper ions to trigger Fenton-like reactions and cuproptosis, while lacking breakthrough carrier architectures or unique therapeutic paradigms. The design remains confined to the simple superposition. In terms of therapeutic characteristics, current systems mostly focus on the synergy between cuproptosis and ferroptosis, or the combination of chemodynamic therapy and photothermal therapy—mechanisms that have been extensively explored in iron-based systems. Consequently, copper-polyphenol systems have yet to establish a truly distinct therapeutic identity that differentiates them from other metal-polyphenol platforms. Further, acute copper toxicity from Cu^+^/Cu^2+^ overload may disrupt iron-sulfur cluster synthesis and essential enzyme function (Liang et al., 2025; Tan et al., 2014)and narrow therapeutic windows between effective tumor killing and systemic toxicity (Liang et al., 2025; Tan et al., 2014). Biological stability challenges include strict copper homeostasis regulation by CTRs and chaperone proteins, potential premature activation in normal tissues, and redox cycle efficiency dependence on intracellular antioxidant levels (An et al., 2024; Chen Y. et al., 2024; Kim et al., 2008; Liang et al., 2025; Tan et al., 2014; Zhang Y. et al., 2024). Clinical translation faces substantial obstacles including undefined regulatory pathways, lack of validated biomarkers for patient stratification, individual variations in copper metabolism and limited long-term toxicity data (An et al., 2024; Chen Y. et al., 2024; Kim et al., 2008; Liang et al., 2025; Tan et al., 2014; Zhang Y. et al., 2024).

### 4.3 Manganese-polyphenol nanomedicines

Manganese-polyphenol coordinated nano-platforms represent an emerging yet unexplored field of cancer therapeutics, leveraging manganese’s Fenton-like catalyst and an indispensable cGAS activator for STING pathway-mediated immunotherapy (Cai et al., 2025; Hart et al., 2015; Horning et al., 2015; Liu et al., 2025; Lv et al., 2020; Tang et al., 2022; Wu et al., 2022; Ye and Kim, 2016; Zhang et al., 2022; Zhang WX. et al., 2024). However, Mn-based counterparts remain extremely scarce with only four representative examples in oncology applications (Cai et al., 2025; Liu et al., 2025; Wu et al., 2022; Zhang WX. et al., 2024). These systems offer distinctive advantages including Mn^2+^-specific cGAS activation for potent immunotherapy (Cai et al., 2025; Liu et al., 2025; Lv et al., 2020; Zhang WX. et al., 2024), multi-modal synergies combining CDT, PTT, gas therapy, and immune activation (Zhang WX. et al., 2024), and excellent biocompatibility from natural polyphenols and albumin components (Cai et al., 2025; Wu et al., 2022).

In manganese-polyphenol systems, researchers have expanded the functionality of traditional metal-polyphenol networks by introducing amino acids (glycine) and multiple metals, opening new directions for development. On one hand, Zhang et al. incorporated glycine into the metal-polyphenol network and prepared metal-polyphenol carbon dots (MP-CDs) using gallic acid and glycine as precursors. This material exhibits excellent biocompatibility and strong photothermal performance. Under near-infrared irradiation, it induces tumor cell damage and promotes the release of damage-associated molecular patterns (DAMPs), thereby facilitating dendritic cell maturation and antigen presentation to achieve PTT-immune synergistic treatment, providing novel insights for constructing metal-polyphenol-amino acid composite systems (Wu et al., 2022). On the other hand, Wang et al. further expanded the design space for multi-metal-polyphenol synergistic therapy through the zinc/manganese bimetallic ZIF-8/MnCO@PDA (ZMP) platform. In the tumor microenvironment, Zn^2+^/MnCO coordinates with polydopamine to enhance photothermal effects and generates •OH via Fenton-like reactions. Simultaneously, MnCO decomposes to release CO and Mn^2+^, which combined with oxidative damage activates the STING pathway, achieving CDT-PTT-gas therapy-immune multi-modal synergistic anti-tumor therapy (Zhang WX. et al., 2024). Compared with these designs featuring clear synergistic logic, remaining manganese-polyphenol systems largely remain at the level of simple superposition of multiple therapeutic modules, lacking deep mechanistic integration and structural innovation.

While critical limitations persist. The therapeutic windows between immune activation and neurological damage are unclear (Horning et al., 2015; Zhang et al., 2022). Biological stability challenges include strict manganese homeostasis regulation by MntE efflux pumps and ZIP8/ZIP14/ZnT10 importers (Zhang et al., 2022) and competition with iron transport systems (Ye and Kim, 2016). Clinical translation faces unprecedented obstacles including completely undefined regulatory pathways for absence of neurotoxicity assessment in all studies despite known risks (Cai et al., 2025; Horning et al., 2015; Liu et al., 2025; Wu et al., 2022; Zhang et al., 2022; Zhang WX. et al., 2024), lack of biomarkers for patient stratification and genetic variations in manganese metabolism transporters (Cai et al., 2025; Liu et al., 2025; Wu et al., 2022; Zhang et al., 2022; Zhang WX. et al., 2024). Future efforts must prioritize comprehensive neurotoxicity evaluation and development of predictive biomarkers to navigate the narrow therapeutic window (Cai et al., 2025; Liu et al., 2025; Wu et al., 2022; Zhang WX. et al., 2024).

### 4.4 Calcium and zinc-polyphenol nanomedicines

Calcium, the body’s most abundant mineral and critical second messenger (Monteith et al., 2017; Zheng et al., 2023), has been utilized in metal-polyphenol therapy only as a passive structural backbone, exemplified by PGFCaCO_3_-PEG/Fe-GA frameworks loading cisplatin prodrugs and doxorubicin (Dong et al., 2020; Han et al., 2022). This design leverages calcium’s biocompatibility and pH-sensitive dissolution for drug delivery, but limitations include calcium’s lack of active therapeutic contribution, potential interference with intracellular Ca^2+^ homeostasis (Monteith et al., 2017; Zheng et al., 2023). Biological stability concerns involve premature dissolution in acidic microenvironments and competition with endogenous Ca^2+^-binding proteins (Monteith et al., 2017). Scalability is hindered by precise control requirements for amorphous CaCO_3_ crystallinity and coating uniformity (Dong et al., 2020; Han et al., 2022). Clinical translation faces regulatory uncertainties, absence of predictive biomarkers, and potential systemic disturbances to bone and cardiac function (Dong et al., 2020; Han et al., 2022; Monteith et al., 2017; Zheng et al., 2023). In the future, scientists’ efforts should explore direct Ca-polyphenol coordination to unlock calcium’s intrinsic bioactivity rather than minor roles.

Zinc, the second most abundant trace metal, serves as a critical intracellular second messenger with tightly regulated homeostasis (Inoue et al., 2015; Kumar et al., 2021; Pan et al., 2017; Yamasaki et al., 2007), yet no Zn-polyphenol coordination nanomaterials exist for cancer therapy. Currently, zinc acts only as a passive structural carrier such as ZIF-8 without therapeutic contribution (Zhang LR. et al., 2024), which misses opportunities to leverage zinc’s intrinsic immunomodulator and anti-tumor functions (Pan et al., 2017; Yamasaki et al., 2007). Limitations include uncontrolled Zn^2+^ release disrupting homeostasis and potential neurotoxicity. Clinical translation faces regulatory uncertainties, biomarker absence, and systemic overload risks affecting bone and neurological function (Inoue et al., 2015; Kumar et al., 2021; Pan et al., 2017; Yamasaki et al., 2007). Zn-polyphenol coordination systems are highly anticipated.

## 5 Summary and future perspectives

Polyphenols exhibit potent anti-tumor activity, yet its low bioavailability limits clinical utility (Li et al., 2022; Zhou et al., 2016). Coordination with suitable metal ions overcomes this drawback, yielding stable metal-polyphenol networks that suppress tumor proliferation and metastasis through multiple signaling pathways. These materials are expected to preserve the full therapeutic advantage while additionally serving as a platform to co-load cytotoxic drugs, thereby achieving tripartite synergy among the metal, polyphenol, and chemotherapeutic agent. The phenolic hydroxyl groups of polyphenols coordinate rapidly and under environmentally benign conditions with metal ions to generate metal-polyphenol nanomaterials. These constructs retain the therapeutic activities of their raw materials while synergistically amplifying anti-tumor efficacy. Intriguingly, reaction parameters can be tuned on demand to produce nanodots, nanonets, or other architectures that optimize drug loading and minimize premature release. Above all, metal-polyphenol nanomaterials have been extensively investigated in biomedicine and offer the following advantages: (1) Simple and green fabrication; (2) High biocompatibility and reduced drug toxicity; (3) Tumor-microenvironment responsiveness that promotes targeted drug delivery and controlled release for precise malignant-tumor therapy; (4) functionalization, enabling loading or coating of metabolic inhibitors or chemotherapeutics to construct versatile nano-platforms for multimodal synergistic cancer treatment. However, constrained by the intrinsic properties of the metal ions, current metal-polyphenol anti-tumor systems are almost monopolized by Fe-polyphenol combinations, while the polyphenols are aimed to EGCG, GA and TA. We anticipate that future researchers will decipher the coordination codes of Fe, Cu, Mn and other metals with novel polyphenols, which injects fresh vitality into tumor therapy. Meanwhile, research on novel metal-polyphenol nanomedicines should intensify investigation into the anti-tumor signaling pathways mediated by polyphenols.

In future, a highly promising research direction is the design and construction of more complex and multifunctional metal-polyphenol coordination systems. Current studies have primarily focused on combinations of single metal ions with single polyphenol ligands. Although these have demonstrated good efficacy in tumor therapy, there remains substantial room for improvement in terms of functional integration and synergistic effects. To address this, scientists could break through the limitations of traditional single-component systems and explore diverse coordination structures such as dual-metal-polyphenol, metal-dual-polyphenol, dual-metal-dual-polyphenol, or even multi-metal-multi-polyphenol architectures. More intriguingly, whether metal-polyphenols could coordinate with metal-amino acids to form metal-amino acid-polyphenol nanostructures merits further consideration. This would enable multidimensional therapeutic effects. Specifically, dual-metal centers could confer differentiated catalytic activities or biological functions—for instance, one metal for Fenton reaction and another for targeted recognition or imaging-guided tracking. Meanwhile, dual-polyphenol could integrate the specific advantages, such as one responsible for drug loading and the other enhancing antioxidant or anti-inflammatory properties. The biosafety of metal-polyphenol nanomedicines warrants careful consideration. Current studies lack long-term chronic toxicity data and standardized production protocols, with a narrow therapeutic window between effective tumor killing and systemic toxicity. Prior to clinical application, long-term safety assessments and compliant manufacturing studies must be conducted, markers must be established, and indications must be selected to reduce systemic risks, thereby facilitating the translation of this innovative strategy from bench to bedside. Although the complex systems still face many challenges, they offer considerable scope for innovation to develop next-generation intelligent responsive tumor diagnosis and treatment platforms.
